# How does landscape composition and configuration affect dung beetle communities in Eastern Austrian agricultural landscapes?

**DOI:** 10.1007/s10980-025-02207-9

**Published:** 2025-10-03

**Authors:** Raja Imran Hussain, Benjamin Knittl, Christian H. Schulze, Thomas Frank

**Affiliations:** 1 Institute of Zoology, Department of Ecosystem Management, Climate and Biodiversity, BOKU University, Gregor-Mendel-Straße 33, A-1180 Vienna, Austria; 2 Division of Tropical Ecology and Animal Biodiversity, Department of Botany and Biodiversity Research, University of Vienna, Rennweg 14, A-1030 Vienna, Austria

**Keywords:** Coprophagous beetles, Landscape heterogeneity, Set-aside land, Species richness, Species composition

## Abstract

**Context:**

Understanding the effects of landscape composition on biodiversity is crucial, especially in human-dominated agricultural landscapes. This study focuses on dung beetles, an ecologically significant group, to explore how landscape composition and configuration influences species richness, abundance and community structure of dung beetles in Eastern Austrian agricultural areas.

**Objectives:**

The primary objective of this study is to investigate the relationship between landscape composition and dung beetle communities. Specifically, we aim to determine how different habitat types within agricultural landscapes affect dung beetle species richness, abundance and community structure.

**Methods:**

We sampled dung beetles across 14 study landscapes, each with a diameter of 1 km, varying from homogenous landscapes dominated by annual crops to highly heterogeneous landscapes with diverse habitats such as woodlands, hedgerows, and set-aside land (areas left fallow or uncultivated). The study design focused on comparing dung beetle communities across these varying landscape compositions.

**Results:**

Our results reveal that dung beetle species richness is positively correlated with woodland cover, set-aside land and hedgerow length, while beetle abundance is associated with woodland cover and hedgerow length. Additionally, dung beetle communities were highly nested, with communities from landscapes with less woodland cover nested within those from landscapes with higher woodland cover. This underscores the importance of heterogeneously structured landscapes, such as woodlands, for maintaining diverse dung beetle communities. These findings highlight that a decline in structural diversity, often caused by agricultural intensification, likely reduces the ecosystem services provided by dung beetles.

**Conclusions:**

The study’s findings emphasize the significance of maintaining landscape structural diversity to support dung beetle communities and their associated ecosystem services. Recommendations for landscape management and planning include promoting heterogeneous landscapes with set-aside land to enhance biodiversity and ecosystem functioning in agricultural areas.

## Introduction

Agricultural landscapes are some of the most heavily modified ecosystems by humans. The intensification of agriculture has caused a significant decline in biodiversity (Weibull et al. 2003; Shah et al. 2023; Shi et al. 2024). This loss of biodiversity is concerning because many species provide essential ecosystem services, such as pollination, pest control, and nutrient cycling (Albrecht et al. 2012; Blaauw and Isaacs 2014; Tschumi et al. 2016; Bürgler et al. 2024). To maintain biodiversity in these areas, habitat heterogeneity and the presence of semi-natural habitats are crucial (Haaland et al. 2011; Priyadarshana et al. 2024). However, agricultural intensification, which involves the increased use of fertilizers, pesticides, and monocultures, has simplified habitats and reduced farmland biodiversity (Geiger et al. 2010; Tscharntke et al. 2012; Hussain et al. 2021, 2023). Understanding how landscape composition affects biodiversity is therefore essential for developing effective conservation strategies (Landis 2017; Martin et al. 2019; Shah et al. 2023). Further, landscape configurations, such as the spatial arrangement of habitat patches, also play a crucial role in influencing arthropod communities in agricultural landscapes (Rischen et al. 2023). Landscape configuration can affect the movement, survival, and reproduction of arthropods by altering habitat connectivity and the availability of resources (Fahrig et al. 2011; Duflot et al. 2017).

Dung beetles (Scarabaeidae) are an important group of insects that play a key role in ecosystems. They contribute to soil fertilization, nutrient cycling, seed dispersal, and pest control (Nichols et al. 2008; Torabian et al. 2024). Because they are sensitive to environmental changes, dung beetles are often used as bioindicators of landscape health (Nichols et al. 2007; Gardner et al. 2008). Previous research has shown that land-use intensity negatively impacts dung beetle diversity across different ecosystems, from forests to agroforestry systems and annual crops (Shahabuddin et al. 2005; Harvey et al. 2006Shahabuddin et al., 2010). In agricultural landscapes, woodlands and set-aside lands have been found to support higher diversity in arthropod communities (Drapela et al. 2008; Querner et al. 2013), including dung beetles (Kessler et al. 2011; Schernhammer 2020). Set-aside land refers to areas of agricultural land that are left fallow or uncultivated to promote biodiversity and ecosystem health. These habitats provide important resources, such as food and breeding sites, and act as refuges from the disturbances caused by farming activities (Jeanneret et al. 2003; Woodcock et al. 2005).

This study focuses on dung beetle communities in Eastern Austrian agricultural landscapes, which range from simple, homogeneous croplands to complex, heterogeneous landscapes with diverse habitats. We predict that set-aside lands will have a positive effect on dung beetle species richness and abundance by providing critical refuge, diverse microhabitats, and reduced agrochemical exposure, supporting ecological functions and biodiversity persistence (Babu 2018; Escudero et al. 2025). Additionally, we expect that landscapes with higher structural complexity will support more diverse dung beetle communities (Martin et al. 2019). In contrast, intensively managed agricultural landscapes are likely to have simpler communities that are subsets of the richer communities found in more complex landscapes. These predictions are based on the idea that woodlands and set-aside lands habitats provide critical resources and favorable conditions for dung beetles, which are necessary for their survival and reproduction (Frank et al. 2017; Barretto et al. 2020).

## Methods

### Study area and study sites

**Figure 1.**
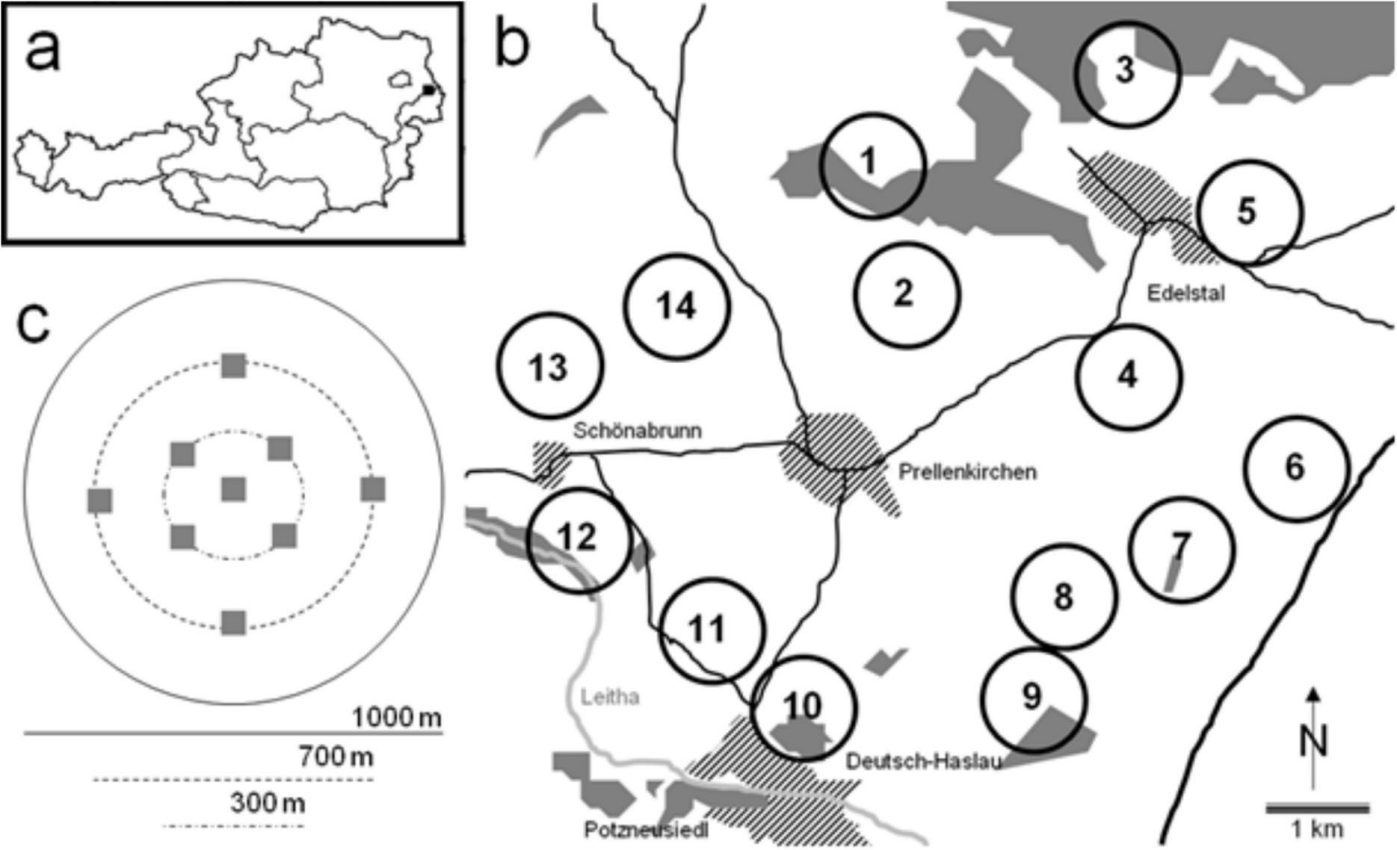
Maps showing (**a**) the locations of the study landscapes in eastern Austria and (**b**) the arrangement of the 14 sampled landscapes (circles; major rivers (pale grey line), major roads (black lines), villages (hatched areas) and all larger woodlands (dark grey areas)). Additionally, (**c**) the spatial distribution of the 9 dung baits (grey squares) placed at the center and at radii of 150 m and 350 m within each study landscape is illustrated.

Dung beetle communities were sampled at 14 study sites in the eastern part of Lower Austria and in the northern part of Burgenland, Austria (Figure 1). The study sites in Lower Austria were located around the villages of Deutsch-Haslau, Schönabrunn and Prellenkirchen, in Burgenland around Edelstal. The entire study area is part of the Vienna Basin, bounded by the hills Spitzerberg and Hundsheimer Berge in the north and by the Pannonian Basin in the southeast. The main soil type in our study region is black soil (http://www.bodenkarte.at/). The main field crops were cereals followed by maize, rape, sugar beet, potatoes, and wine. The mean annual temperature of our study area is 10.0 °C, the mean annual precipitation is 637 mm (measured at Hainburg; http://de.climate-data.org/location/159305/).

All 14 study sites were situated at 130−190 m a.s.l and represented “circular landscapes” (subsequently termed “study landscapes”) with a diameter of 1 km (Fig. 1b-c). These sampled study landscapes are a subset of 29 randomly selected landscapes used in the study by Drapela et al. (2008) and represent a gradient from simple landscapes predominantly characterized by crops (mainly cereals and maize) to landscapes with a high structural complexity and habitat diversity. Structural complexity in these landscapes is characterized by factors such as the mean size of crop fields, the connectivity between habitat patches, and the edge densities of these patches. Landscapes with higher structural complexity typically have smaller crop fields, higher connectivity, and greater edge densities, providing more diverse microhabitats and resources for dung beetles. The following landscape variables were measured and used for the analysis in the present study: set-aside land (areas left fallow or uncultivated), hedgerow length, woodland cover (including both continuous forests and tree lines above a certain threshold, i.e. 0.5 hectares for forests and 5 meters for tree lines), annual crop cover (all in % of total area), and structural landscape diversity (Appendix Table A.1). Structural landscape diversity was quantified using two measures: the mean perimeter area ratio (MPAR) of all land cover patches, which indicates landscape-level geometric complexity or fragmentation, and the number of shapes characterizing points (NSCP) index, which represents landscape complexity (Moser et al. 2002). Land cover types were measured using digital orthophotos and satellite imagery analyzed via Geographic Information Systems (GIS).

**Table 1.** Results of GLMs testing for effects of NSCP, set-aside land, woodland cover and hedgerow length on estimated species richness and abundance of dung beetles

Variables	B	SE	95% CI		Wald Chi-	p
			lower	Upper	*Square*	
Species richness						
(Intercept)	15.39	9.54	− 3.32	34.09	2.60	0.107
log (NSCP)	− 4.01	2.23	− 8.43	0.32	3.31	0.069
arcsin (set-aside land)	30.30	10.76	9.22	51.39	7.93	0.005
arcsin (woodland cover)	47.39	10.45	26.90	67.88	20.55	<0.001
log (hedgerow length + 1)	2.08	0.90	0.32	3.84	5.38	0.020
Abundance						
(Intercept)	− 76.831	144.34	− 359.73	206.07	0.28	0.595
log (NSCP)	− 3.77	33.73	− 69.87	62.34	0.01	0.911
arcsin (set-aside land)	− 90.70	162.70	− 409.60	228.19	0.31	0.577
arcsin (woodland cover)	418.15	158.12	108.23	728.07	6.99	0.008
log (hedgerow length + 1)	29.06	13.57	2.46	55.66	4.59	0.032

### Sampling and identification of dung beetles

To account for seasonality, dung beetles were sampled in all landscapes four times in May, July, September and October 2007, respectively. During all sampling periods a total of nine dung baits (each consisting of 250 g of fresh horse dung) were exposed in each landscape in similar habitats for three days. At each study landscape one dung bait was placed in its centre, while the others were located at a radius of 150 m (4 traps) and 350 m (4 traps) around it. This spatial design of trapping should enable to sample the majority of dung beetle species of the study landscapes. The used dung was always from the same two horses; none of those received any medical treatment during the whole time, which potentially could have affected the results (e.g. Römbke et al. 2007). While the landscape variables were measured within a 500 m radius based on the center dung bait, we acknowledge that this may not fully capture the surrounding landscape effects for all 9 baits.

Baits were re-collected after three days from the field. Additionally, the soil surface below the baits was collected to a depth of 5–10 cm to include beetles feeding on dung particles buried into the soil beneath the dung pats. During the four sampling periods (May, July, September, October) dung pats were always placed at the same positions. To assure that dung baits can be re-located, locations of dung pats were marked with coloured wooden sticks. Therefore, only 3–4 dung baits out of 126 (2–3%) were lost during each sampling period. To extract the beetles from the dung and the collected soil, a Berlese funnel was used (Southwood 1978). Two 500 W halogen headlights and a 200 W infrared lamp were used to create a gradient of decreasing dung humidity towards the bottom of the dung. Trying to escape the decreasing humidity, beetles crawl deeper into the funnels and fall into jars filled with a killing agent (70% ethanol). To avoid the escape of beetles, the top of the funnel was closed with gauze. To validate this method, dried out dung was investigated for remaining beetles, which demonstrated that nearly 100% of the beetles were successfully extracted from the dung. All caught beetles were preserved in 70% ethanol. In this study only coprophagous beetles belonging to the family Scarabaeidae were considered. Beetles were identified according to Bunalski (1999) and Klausnitzer (1994a, b a, b) and by using a reference collection based at the Department of Botany and Biodiversity Research, University of Vienna.

### Data analysis

To estimate the total species richness for each landscape, we used the Jackknife 2 (Jack2) estimator, which is known for its precision and robustness in richness estimation (Walther and Moore 2005). Both species richness (estimated by Jack2) and total beetle abundance were tested for normality using the Kolmogorov-Smirnov test, and neither deviated significantly from a normal distribution (p > 0.20). Therefore, we applied generalized linear models (GLMs) with a normal error distribution and an identity link function to assess the effects of landscape variables on dung beetle richness and abundance. Generalized linear models (GLMs) were constructed using all possible combinations of these variables. The significance of each term was tested through the deviance that the removal of the term adds to the model. Akaike’s Information Criterion (AIC) was used to select the best models, with the lowest AIC indicating the best fit. The effect of MPAR (Mean Perimeter Area Ratio) on dung beetle species richness and abundance was statistically tested using generalized linear models (GLMs), with MPAR included as an independent variable.

Due to a significant negative correlation between crop cover and woodland cover (r = − 0.81, p < 0.001), these variables were not included together in a single model to avoid multicollinearity. Instead, separate models were constructed for each variable.

To analyze the nestedness of species communities, we calculated Monte Carlo-derived probabilities for presence-absence matrices being random. Nestedness was tested by comparing the system temperatures of these matrices with the average temperature of 1000 randomized matrices using the binmatnest algorithm (Rodríguez-Gironés and Santamaría 2006). Spearman rank correlations were calculated to test for relationships between the ranking of sampled landscapes in the presence-absence matrix packed into a state of maximum nestedness and all landscape variables. Subsequently, false discovery rate (FDR) transformations were applied to correct for bias caused by multiple testing (Pike 2011).

Species composition similarity between the 14 study sites was quantified using Bray-Curtis similarities, calculated from square-root transformed abundance data to reduce the influence of dominant species (Clarke and Warwick 2001). These similarity relationships were visualized using non-metric multidimensional scaling (NMDS) ordination. A stress value of < 0.20 was considered to adequately represent the similarity relationships (Clarke 1993). We performed Spearman rank correlations to evaluate the effects of landscape structure on species composition by testing the relationships between NMDS axis scores and landscape variables. This approach allowed us to identify significant correlations between species composition and landscape variables, which are displayed in Figure 3. The landscape variables included in the analysis were annual crops, set-aside land, woodland cover and hedgerow length. All statistical analysis were performed in Statistica version 7.1.

## Results

In total 23 scarabaeid dung beetle species were recorded. Of the 1427 specimens sampled, the majority belonged to the genera *Onthophagus* and *Aphodius* with 714 and 710 individuals and 9 and 11 species, respectively. In addition, singletons of *Oxyomus sylvestris*, *Rhyssemus germanus* and *Trypocopris vernalis* were collected.

### Effects of landscape parameters on species richness and abundance

**Figure 2.**
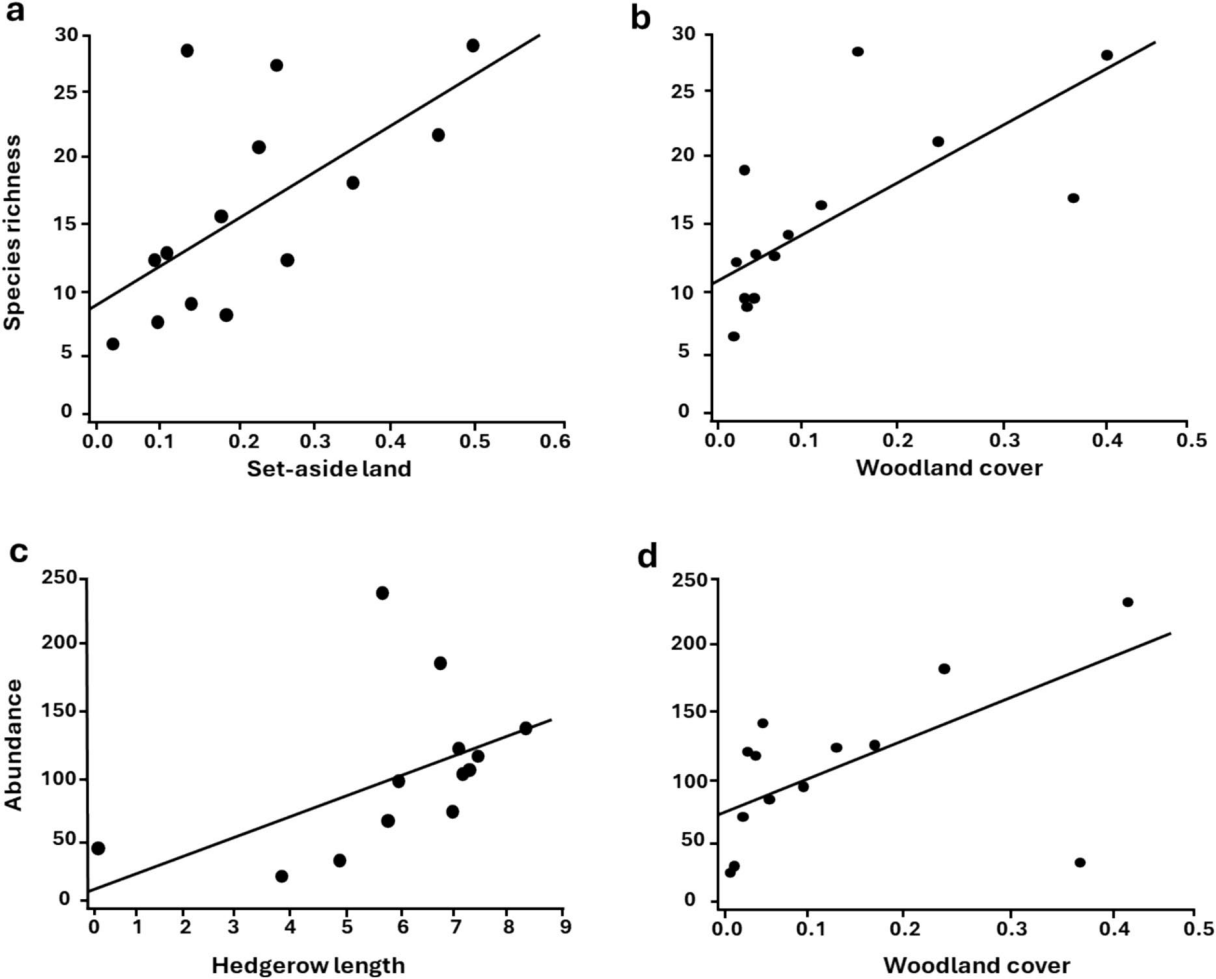
Linear regression showing significant (p = 0.05) relationships between species richness (estimated by Jack2) and abundance, and the habitat variables: (**a**) set-aside land, (**b**, **d**) woodland cover, and (**c**) hedgerow length, as predicted by a GLM (with normal error distribution and identity link-function).

We observed a positive effect of woodland cover and hedgerow length on species richness and abundance. Further, a strong positive effect of set-aside land on species richness (but not abundance) was detected. NSCP did not show any clear effect on neither richness nor abundance of beetle communities (Table 1, Figure 2).

### Similarity and nestedness of species communities

Occurrence of dung beetle species in the 14 sampled landscapes was not random but proved to be highly nested (binmatnest: matrix T = 14.70, p < 0.001). The rank of landscapes in the presence-absence matrix packed into a state of maximum nestedness was closely related to woodland cover, but not to NSCP, set-aside land and hedgerow length (Appendix Figure A.1). The relation with crop cover did not remain significant after adjusting it for FDR (Table 2).

**Table 2.** Results of Spearman rank correlations to test for relationships between the ranking of sampled landscapes in the presence-absence matrix packed into a state of maximum nestedness (Figure 3) and all landscape variables

Variables	*r*_*s*_	*p*	*FDR adjusted p*
log (NSCP)	− 0.16	0.5829	0.7286
arcsin (crop cover)	0.59	0.0265	0.0662
arcsin (set-aside land)	− 0.43	0.1258	0.2097
**arcsin (woodland cover)**	− 0.74	0.0023	0.0115
log (hedgerow length + 1)	− 0.09	0.7705	0.7705

Habitat variables printed bold remained significant after adjusted for false discovery rate (FDR).

**Figure 3.**
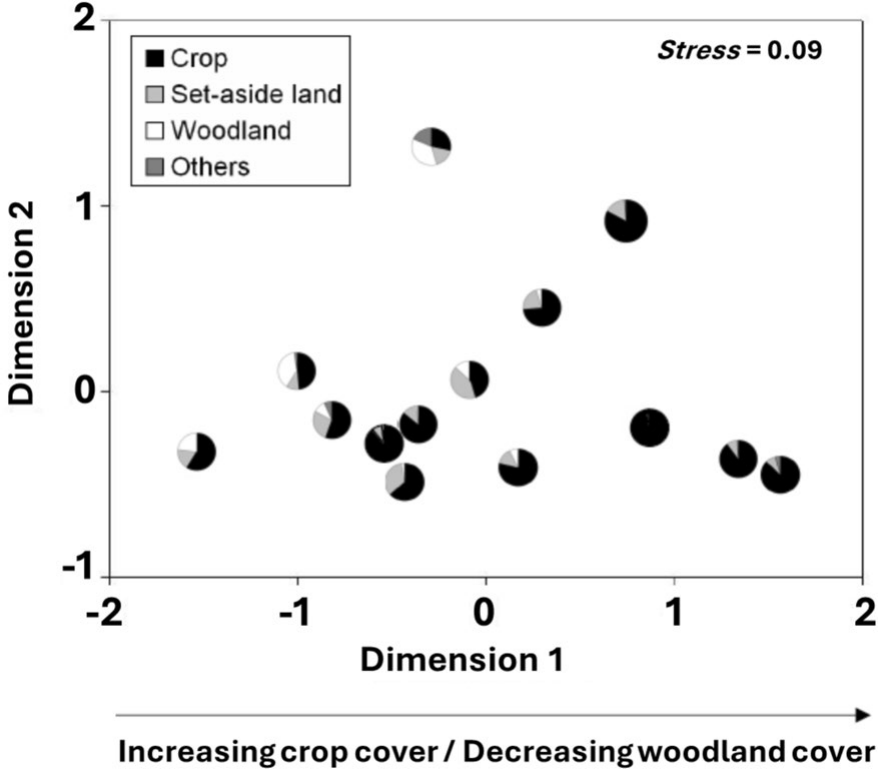
NMDS plot based on Bray-Curtis similarities (square-root transformed abundances) of dung beetles in 14 landscapes. Dimensions 1 and 2 are shown. Pie charts at the plot centers show the percentage cover of crop, set-aside land, woodland, and other habitats. Dimension 1 values were significantly correlated with woodland and crop cover.

Similarity relationships (quantified by Bray-Curtis similarities using square-rooted transformed abundances) between dung beetle communities of the 14 landscapes were visualized in a 2-dimensional NMDS plot (Figure 3). The plot as well as correlations between Dimension 1 values and landscapes variables indicate that changes in species composition are related to woodland cover (r_s_ = 0.35, p = 0.025) and crop cover (r_s_ = 0.37, p = 0.022). No relationships between set-aside land and MPAR and Dimension 1 values were found and none of the correlations between landscape variables and Dimension 2 values achieved a significant level (all p values > 0.1).

## Discussion

In this study, most species were caught in landscapes with a high proportion of woodlands and hedgerow length. Areas with a high land-use intensity were characterized by an extremely depauperate dung beetle fauna. Such homogeneous landscapes dominated by habitats characterized by high land-use intensity also did not contribute to the overall species richness. Their species communities proved to be mainly subsets of landscapes with a higher proportion of woodlands and set-aside lands.

Differences of dung beetle communities between sampled landscapes may be particularly shaped by the three important factors (1) food availability, (2) habitat microclimate, and (3) soil structure (Silva and Hernández 2014). All three factors may be affected by changes of landscape composition predominantly related to land-use intensity, as intensive land use often reduces the diversity and abundance of dung-producing animals, which are essential for dung beetle sustenance (Frank et al. 2017). Additionally, intensive land use can modify habitat microclimate and soil structure by changing vegetation cover and soil compaction (Kuntzman and Brom 2024), respectively, thereby impacting the microhabitats that dung beetles rely on for breeding and feeding (Barretto et al. 2020). Most dung beetles are considered opportunistic and use a wide variety of excrement types without much discrimination (Tonelli et al. 2021). Furthermore, many species use both dung and carrion (Gimenez et al. 2021). The most important dung producing mammals in our study area are wild boar (*Sus scrofa*) and roe deer (*Capreolus capreolus*) (own observations). Both use woodland and set-aside land for foraging, resting and hiding. Therefore, landscapes with lower woodland and set-aside land are characterized by lower densities of these large mammals (Thurfjell et al. 2009; Schwegmann et al. 2023). Together with other herbivorous and carnivorous vertebrates, their dung represents the most important natural food source for dung beetles in agriculturally used areas. Empirical evidence indicates that a large and diverse mammal fauna is crucial for the maintenance of a large and diverse dung beetle fauna (Nichols et al. 2008). Consequently, a high abundance and diversity of dung producers should positively affect the richness of dung beetle communities. Dung beetles partition their food and breeding sources according to their physico-chemical attributes. These include water content, fiber size, dropping size, and nutritional quality (Tshikae et al. 2008). While the latter three parameters do exclusively depend on the type of dung producer, water content depends predominantly on the microclimate which can change dramatically between intensively used open areas (such as farmland), and particularly woodland (Nervo et al. 2021). Dung droppings in woodland and set-aside land need more time to dry out. Hence, they are longer usable and detectable for most dung beetles than droppings exposed in agricultural areas, because they are better shaded and are sheltered from wind and sun by trees and a high grass/herb layer, respectively. Therefore, the microclimate of set-aside land and woodland should have a positive effect on faunal richness of dung beetles (Righi et al. 2018; Davis et al. 2013).

Additionally, soil type and structure can have prominent effects on the occurrence of dung beetle species. Both can be important for tunnel structure and depth, and consequently for the breeding success (Bertone et. al. 2006). Unlike agriculturally used soils, forest and fallow soils offer better aeration and more structures for the protection against desiccation. Likewise, in crop areas, intensive rain is a bigger threat to the breeding chambers (Sun et al. 2023). The reason why less species are present in areas that are intensively agriculturally used could be the recurrent disturbance of the soil because of tillage (Muoni et al. 2019).

Furthermore, the widespread use of insecticides, herbicides, fungicides, and anthelmintics can be responsible for a decreasing dung beetle population (Torabian et al. 2024). Therefore, areas such as set-aside land and woodland, which are not directly affected by the applications of such chemicals, may represent refuges maintaining higher local species richness. From these “habitat islands” dung beetle species may be also able to re-colonize the adjacent farmland. In this study, increasing diversity and spatial heterogeneity of habitats quantified by NSCP and MPAR did not show any impact on species richness. Dung and carrion are ephemeral resources with a patchy distribution of space and time (Butterworth et al. 2023). Although vision is essential for navigation when flying in search for fresh dung, dung beetles primarily locate their food and breeding resources by olfactory cues that are provided by the many chemical compounds that are released from dung as volatiles, either in small or large quantities (Tshikae et. al. 2008). Dung beetles can detect food and breeding resources from more than at least 100 m. They are mobile and able to cross longer distances between different dung pats (Huerta et al. 2010). Hence, the structure and complexity of a landscape might be less vital for them than for other less mobile insects.

## Conclusions

This study underscores the importance of woodland, hedgerows and set-aside land, in maintaining dung beetle diversity within agricultural landscapes. These habitats provide essential resources and favorable conditions that support diverse dung beetle communities. Further, preserving and integrating these habitats into agricultural areas is crucial for enhancing biodiversity and the ecosystem services provided by dung beetles, such as nutrient cycling and soil health. Conservation strategies should prioritize the protection and restoration of these habitats to mitigate the adverse effects of agricultural intensification. By maintaining landscape heterogeneity, it is possible to create a more resilient agricultural ecosystem that supports biodiversity and agricultural productivity. Hence, the study highlights the need for a balanced approach to land management that considers both agricultural needs and biodiversity conservation.

## Figures and Tables

**Figure 4.**
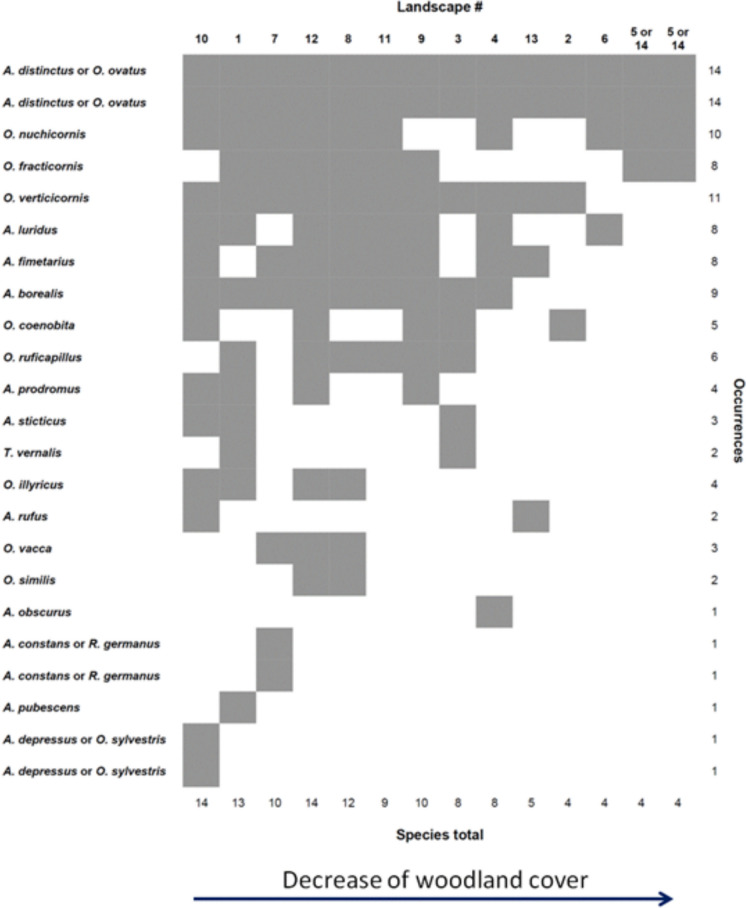
Presence-absence matrices of dung beetles recorded at 14 study sites packed into a state of maximum nestedness. Study sites are in columns, species are in rows. Grey cells indicate occurrences of individual species at the sampled sites. Landscape number correspond to the numbers provided in Figure 1.

## Data Availability

The datasets collected and analyzed during the current study are available from the corresponding author on reasonable request.

## Appendix

See Table

**Table 3:** Measured landscape variables

Landscape	MPAR	Crop cover (%)	Set-aside land (%)	Woodland cover (%)
1	0.138	39.60	9.70	33.10
2	0.246	55.10	19.70	4.20
3	0.169	61.90	11.70	1.40
4	0.240	64.10	8.50	0.50
5	0.133	67.90	1.70	0.10
6	0.068	59.30	13.10	7.40
7	0.170	49.10	29.10	2.70
8	0.044	12.00	11.30	1.90
9	0.122	41.20	38.50	14.00
10	0.183	47.50	17.30	21.40
11	0.103	64.10	8.20	2.20
12	0.171	44.20	23.90	10.20
13	0.088	60.40	14.60	0.20
14	0.056	62.90	7.40	0.30

*MPAR* Mean perimeter area ratio of all land cover patches, *NSCP* Number of shape characterizing points

3, Fig. 4
